# A Methodology for Validating Safety Heuristics Using Clinical Simulations: Identifying and Preventing Possible Technology-Induced Errors Related to Using Health Information Systems

**DOI:** 10.1155/2013/526419

**Published:** 2013-03-31

**Authors:** Elizabeth Borycki, Andre Kushniruk, Christopher Carvalho

**Affiliations:** School of Health Information Science, University of Victoria, Victoria, BC, Canada V8W 3P5

## Abstract

Internationally, health information systems (HIS) safety has emerged as a significant concern for governments. Recently, research has emerged that has documented the ability of HIS to be implicated in the harm and death of patients. Researchers have attempted to develop methods that can be used to prevent or reduce technology-induced errors. Some researchers are developing methods that can be employed prior to systems release. These methods include the development of safety heuristics and clinical simulations. In this paper, we outline our methodology for developing safety heuristics specific to identifying the features or functions of a HIS user interface design that may lead to technology-induced errors. We follow this with a description of a methodological approach to validate these heuristics using clinical simulations.

## 1. Introduction

Health information system (HIS) safety has emerged as growing concern around the world for clinicians, regional health authorities, and governments who are modernizing health care through information technology. Internationally, we have seen the number of studies documenting the existence of technology-induced errors grow [1, 2]. Governments in Canada, the United States, and Europe have begun to address this issue from a policy perspective in the United States, Canada, Australia, and the European Union [3]. Researchers are now examining this emerging and critical issue by developing methods that can be used to identify potential HIS safety issues prior to implementation [4]. In this paper, we outline a novel, evidence-based methodological approach that can be used to develop and validate safety heuristics and can be used to evaluate the safety of HIS user interface features or functions before a HIS is implemented in a healthcare setting. We begin our journey by outlining the definitions for a technology-induced error and HIS safety as well as some of the research in this area documenting these types of errors. 

## 2. Review of the Literature

Technology-induced errors have arisen as a significant international issue [5]. In several countries around the world (e.g., Australia, Canada, China, and the United States) reports are emerging and research has documented the existence of instances where HISs have been found to contribute to patient harm and death [5–7]. These types of errors have been referred to in the literature as “technology-induced errors.” Technology-induced errors can be defined as those sources of error that “arise from (a) the design and development of technology, (b) the implementation and customization of a technology, and (c) the interactions between the operation of a technology and the new work processes that arise from a technology's use” [8, page 154]. Research involving technology-induced errors focuses upon the complex interactions between HIS and health professionals [9]. Historically, information system designers have developed technologies that can automate work processes in industries such as banking, where there is a lower chance that an information system will lead physical harm or death. 

Today, we have seen an increase in the number of software companies who are designing information systems for healthcare [10, 11]. Along with this, the number of published accounts of how HISs are involved in technology-induced errors leading to human death and disability has also increased [1, 5–7, 12–16]. For example, there have been several studies that have taken place at a regional, statewide, or national level involving incident reporting databases. These studies have found there to be instances where HISs have contributed to potential and real harms/deaths in patients [4, 7, 17]. For example, Magrabi and colleagues in their analysis of data from an “Advanced Incident Management System (AIMS)” used by 50% of Australian states and territories found there to be 99 incidents where computer problems affected patient safety. Most of the incidents either were machine-related (i.e., 55%) or had their origins in human computer interaction (i.e., 45%). Machine-related incidents included general technical issues such as computer systems being down or too slow, problems associated with accessing the system, the software being unavailable, software issues emerging during use, or data being lost [7, page 666]. Human computer interaction-related incidents included those issues associated with inputting data, transferring information, and errors associated with receiving/obtaining information from software. The authors reported that 38% of the incidents had some consequence, but no harm had occurred to a patient [7, page 663]. In another study conducted using the United States Food and Drug Administration incident reporting system (i.e., MAUDE), using data collected over a two-year period, researchers found that 96% of technology-induced errors were machine related while 4% arose from human-computer interaction problems. In this work, 11% of technology-induced errors were associated with patient harm and death [5]. Samaranayake et al. [17], in a 5-year analysis of data from a large tertiary care hospital, found that 17.1% of all reported incidents involved technology-related errors. Many of these incidents had their origins in interface design and socio-technical issues [4, page 828]. Some of these errors lead to wrong drug errors, dosage errors, duration errors, and instruction errors [17, page 831]. In summary technology-induced errors have emerged as a significant concern internationally as large-scale studies of incident reporting systems are identifying the presence of these types of errors. 

HIS safety or “activities that seek to minimize or to eliminate hazardous conditions that can cause bodily injury” arising from the use of information systems and technologies have emerged as a new area research [18]. Some of this work has begun to examine the relationship between user interface design and HIS safety. According to the research literature, HISs with effective user interface designs have the ability to improve the quality of health professional decision-making and to reduce the number of medical errors made [13, 14, 19]. Alternatively, HISs with poor user interface designs may lead to health professional user errors (i.e., technology-induced errors) once released for use in patient care [1, 5]. User interface designs themselves can range in terms of their ability to promote safety and detract from safety (with some systems having both safe and unsafe features or functions) [20]. User interface design features refer to the “structure, form, or appearance” [21] of an interface design while software functions refer to activities that computers support or undertake [22]. It is difficult for software designers to design and develop HIS user interfaces for complex environments such as those found in healthcare. In the design and development process, the unique characteristics of healthcare work settings need to be accounted for in the user interface itself [10, 11]. Furthermore, the dynamic nature of these healthcare environments, the frequency of occurrence of urgent, atypical events, and the presence of uncertainty for users (e.g., physician) place significant demands upon HIS user interfaces [1, 23]. Yet, there is a need for research that outlines how user HIS interface designs can support complex work activities while at the same time not introducing new types of technology-induced errors [1, 24]. This is especially the case in healthcare. 

In some communities, this has emerged as a barrier to HIS adoption and has initiated discussions and publications regarding software developer and vendor blame from a legal perspective [25, 26]. Others have determined that software vendors and their employees can be held legally accountable for harms arising from poorly designed and implemented software [1, 23, 25, 26]. These publications have led some country and government organizations to begin the process of monitoring and regulating software developed in health care (e.g., United States, European Union) [3, 6].

Researchers have recognized the need to design and develop HIS user interfaces for safety (i.e., user interface designs that prevent technology-induced errors). Researchers are using different approaches to address this issue from a software design, development, and testing perspective [1, 10–13, 23, 25]. One approach that is increasingly being discussed is the use of evidence-based or research-informed approaches to developing safety heuristics and testing that can be used to identify HIS interface features or functions that may lead to technology-induced errors [15, 23, 27, 28]. Health informatics professionals and software engineers are beginning to examine the use of differing evidence-based approaches to user interface design (i.e., evidence-based software engineering) in medical device design (e.g., intravenous pumps) and how they might apply to healthcare. Less has been done focusing on software designed specifically for healthcare settings (e.g., physician order entry, medication administration systems, electronic health records, electronic medical records, and disease management systems) [15, 29]. However, despite the potential importance of these approaches, the development and empirical validation of such evidence-based safety heuristics has remained to be more fully explored [19]. Recent work by Baylis et al. [14] suggests that Neilsen's heuristics alone cannot be used to assess software for safety. As well, medical device testing approaches cannot be easily applied to software IS interface design due to the nature and purpose of their use [14, 15, 29, 30]. Yet, user interfaces for complex software systems also need to have their safety assessed using evidence-based, validated heuristics [1, 12, 23]. This is especially necessary as errors arising from HIS use in healthcare may lead to human harm (i.e., death, disability, or injury) [6, 31, 32], and the costs of fixing the unsafe software are much lower to vendors than the healthcare costs associated with treating individuals who are harmed by the software [14].

## 3. Methodology

**Figure 1:**
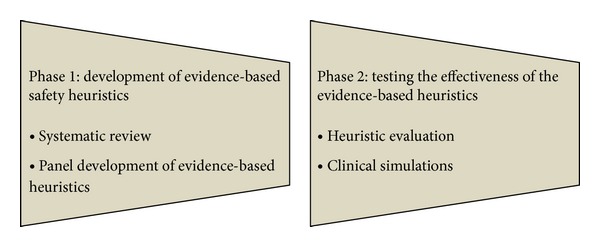
Phase of the methodology for developing and validating safety heuristics.

In this section of the paper, we discuss our methodology for developing evidence-based heuristics and validating them using clinical simulations (see Figure 1). We outline this work (which is done in a series of phases) here in after (see Figure 1).

### 3.1. Phase 1 (Development of Evidence-Based Safety Heuristics)

 A set of evidence-based heuristics for software application safety were developed in a series of stages. Initially, our work on HIS safety consisted of a systematic review of the medical literature in the area of technology-induced errors and healthcare. This was followed by expert panel development of evidence-based heuristics and initial work in conducting clinical simulation testing using simulations to determine if the heuristics could reduce the number of technology-induced “near misses” (i.e., slips) and “errors” (i.e., mistakes) made by health professional users [1, 23, 33]. 

*Systematic Review.* In the first phase of our work, we developed evidence-based, safety heuristics after conducting a systematic review of the literature. The review was undertaken in three stages [1, 10–12, 23]. 

*Stage 1: Key Research Questions.* Our systematic review aimed to answer the following research questions.

1. What are the specific user interface design features or functions that lead to user “near misses” in HIS such as medication administration and physician order entry systems?
2. What are the specific user interface design features or functions that lead to user “errors”?
3. What user interface design features or functions that can lead to “severe” errors (i.e., resulting in human death, disability, and injury)?
4. What is the nature of the situational context where the software application was used and lead to a user “near miss” or “technology-induced error”?

*Stage 2: Preliminary Search for Articles.* In stage 2, we searched the existing literature. An initial search of Medline was conducted using the following keywords: “informatics medical errors”, “ informatics induced errors”, “technology-induced errors”, “technology facilitated errors”, and “computerized physician order entry errors” [23]. The titles and abstracts of articles that were returned from the search were recorded for further analysis in Stage 3. A total of 213 articles were returned from the search. 

*Stage 3: Comprehensive Review of Articles Meeting Criteria.* In stage 3 we selected full-text, English language articles for full review from the initial search that met the following criteria for the study: (1) a technology-induced error was described, (2) a methodology was described for testing for technology-induced errors, or (3) user interface features or functions were described that lead to a “near miss” or “error” [23, 34, 35]. Once the initial search was completed (in stage 2), two health informatics expert reviewers reviewed the citation titles and abstracts independently. Then, the reviewers met to resolve any differences or disagreements between their selections of citations. Once a final set of citations was selected by the researchers, full-text versions of the articles were obtained, and the reviewers again independently reviewed the full-text articles and met to resolve any differences. A total of 10 articles (out of the 213 articles identified in the preliminary search) met the criteria and were selected for full review. A final set of articles were reviewed independently by each reviewer. The following data were extracted: author, year of publication, sample, methodology, and relevant findings (i.e., nature and type of each technology-induced error described in the paper, implications of the error) [12, 23, 27, 29, 34, 36, 37]. The final set of articles were critically appraised for their strengths, weaknesses, and quality [12, 23, 27–29, 34, 36, 37]. The extracted data were then placed into a database. The two reviewers then met again and resolved any disagreements that arose following their assessments of the articles [38]. Lastly, the data extracted from the articles were entered into a table and were analyzed for study characteristics, study designs, and results [23, 34]. 

*Panel Development of Evidence-Based Heuristics.* Following this, a panel of three health informatics, human factors experts were brought together to develop a set of evidence-based HIS safety heuristics. The role of the panel was to develop recommendations based on the systematic review. This involved considering each technology-induced error that was identified in the papers obtained during the searches, followed by a drafting of heuristics that could be used to mitigate these errors (e.g., from an article that indicated that a lack of an emergency override in a HIS led to a technology-induced error; a heuristic dealing with need for inclusion of emergency override capability was proposed). Three human factors experts participated in this evidence-based, heuristic development. The members of the panel had multidisciplinary backgrounds (i.e., computer science, health informatics, clinical) and human factors expertise in healthcare [23, 34]. In the following, we describe the work in more detail.

Data were extracted from relevant studies (see Section 3.1 Systematic Review) and were presented at a panel meeting [39]. The evidence collected in the systematic review was presented to panel participants such that the designs and results of the studies could be easily reviewed (i.e., via summary tables) [23, 34]. The experts reviewed the information in the summary tables and categorized the evidence according to the effectiveness of the study designs as this approach is supported by empirical research [34, page 13]. The expert panel were then used to interpret the evidence and determine its applicability to the development of heuristics (i.e., could the findings be developed into a heuristic that could be used in a usability inspection or cognitive walkthrough). 

If there was agreement among expert panel members, then each finding was discussed and a corresponding heuristic was developed. As each study finding was considered, previously developed heuristics were evaluated to determine if they could be effectively used to detect the technology error that was identified in the study. If a previously developed heuristic applied, then no new heuristic was developed. If no heuristic applied, then a new heuristic was developed. This was done until the review of the studies was completed. All differences of opinion regarding heuristic development and wording were resolved through discussion until unanimous agreement was reached. When the panel completed their work, 38 heuristics emerged.

The heuristics were then analyzed using a content analysis approach. Content analysis was employed as it provides a method for obtaining an objective and qualitative description of the content of text [40]. As each heuristic was composed of text, the text within the heuristic was coded for themes. The panel collectively analyzed the textual components of each of the heuristics based on their knowledge of the human factors literature. This is consistent with the development of themes in content analysis [40]. The textual content of each heuristic was analyzed for themes and coded as falling under a theme or requiring a new theme. If a new theme emerged, when analyzing the heuristics, then it was defined and the heuristic was coded using the new theme. With each heuristic that was considered, the panel members first attempted to code the heuristic with previously developed themes. If the theme did not apply, then a new theme was developed, defined, and applied to the heuristic. This was done until all of the heuristics were analyzed. Four usability themes emerged: content, workflow, functional, and safe guard issues (see Table 1 for themes, definition of each them and corresponding number of heuristics that were categorized under each theme; see Table 2 for examples of developed heuristics for each theme) [23]. All differences in expert panel opinion regarding the coding of heuristics according to specific themes were resolved through discussion until unanimous agreement was reached by the panel. Once the safety heuristics had been developed by the expert panel, the heuristics were disseminated to representative users (i.e., health informatics professional, software developers, and designers of HIS). 

### 3.2. Phase 2 [Testing the Effectiveness of the Evidence-Based Safety Heuristics]

In Phase 2 of our research we assessed the effectiveness of the evidence-based, safety heuristics. In this phase of this research, we tested these evidence-based safety heuristics by conducting a heuristic evaluation where a user interface of a HIS (i.e., an electronic health record system) was inspected. This involved a human factors analyst inspecting the user interface of a HIS to identify and predict potential user error [23]. Following this inspection, clinical simulation testing could be carried out to determine the ability of the safety heuristics to predict errors. 

*Heuristic Evaluation.* In this phase of the research, the safety heuristics developed in Phase 1 were used to inspect a HIS user interface in order to identify and predict potential user errors (i.e., by identifying instances of error prone HIS design based on the heuristics). This was done to determine if usability inspection applying safety heuristics could predict error facilitating aspects of HIS interface designs (as would be identified in subsequent clinical testing involving human subjects interacting with the HIS in realistic simulated conditions). The inspection was conducted by a trained human factors expert who independently analyzed the HIS's user interface for potential technology-induced errors. The approach extended the work of human factors experts in pilot testing and conducting heuristic evaluations of user interfaces involving the application of heuristics specifically aimed at identifying safety issues that may be related to usability problems [23]. This approach extended heuristic evaluation [41, 42] to include analysis of HIS user interface features or functions that could affect software safety [23]. This is also in line with Nielsen's recommendation that category-specific heuristics should be developed that apply to specific classes of products, processes, and domain areas to supplement general usability heuristics [41].

Our findings suggested that not all heuristics could be applied using a traditional heuristic evaluation approach. Two workflow, six content, two functional, and three safeguard heuristics could be applied to the heuristic evaluation of a electronic patient record (during our testing of the heuristics). Twenty-five of the 38 heuristics could not be applied by an analyst conducting a traditional usability inspection. Instead, we determined that the heuristics needed to be tested in the context of a clinical simulation where scenarios that are representative of real-world environments could drive the safety testing [23]. We observed that the safety heuristics could be most readily applied to static features of an electronic patient record user interface using heuristic evaluation. Dynamic features or functions of the interface could only be tested by using clinical scenarios to drive the inspection, or alternatively by having representative users performing representative tasks in a representative setting. Here, the panel identified the need to use clinical simulation to test the remaining safety heuristics. 

*Clinical Simulations.* Clinical simulation testing is the next step in this work. Clinical simulation testing can be defined as the process of observing representative human users carrying out realistic, complex, and representative tasks in the context of a real-world setting. Here, the researchers will undertake clinical simulation testing to determine the ability of the safety heuristics developed in Phase 1 to predict technology-induced errors (arising from interface designs and HIS-system-related workflows). The clinical simulation-based testing takes place in a realistic environment and involves undertaking user testing of complex HIS interfaces in that environment [43]. HIS user interfaces have been tested in simulated, complex settings, representative of real-world environments where HISs are used (i.e., healthcare). Researchers have used the approach to study usability and workflow emerging from HIS and their impacts on health professional work. Clinical simulation testing of the interface design features or functions involves testing HIS interfaces that have been implicated in human death, disability, and injury (i.e., drawn from the systematic review and reports) in order to increase the likelihood of observing errors during the testing sessions. In clinical simulations, human factors experts are used to validate the ecological validity of the clinical simulation, laboratory environment [44]. Typically, clinical simulation testing of HIS features or functions involves 10–20 representative users. In clinical simulation, users are asked to carry out representative tasks in response to several routine, atypical, urgent, nonurgent, and complex work scenarios. In order to determine when a technology-induced error may be likely to be observed, this work includes testing users in situations that have been reported to be associated with a technology-induced error as reported in the literature [1, 12, 44–46]. In clinical simulations, users are asked to “think aloud” while they perform both routine and complex tasks (which may be associated with a higher user error rate) involving HIS [1, 38, 44]. Audio data from the “think aloud” and video data of the users interacting with the HIS are recorded using a video/audio recorder. Computer screen recording data are also collected as users perform tasks involving a HIS (using computer screen recording software). 

Analysis of clinical simulation data involves fine-grained analysis of video, audio, and computer screen data so that technology-induced errors are captured [1, 24, 30, 45]. Data from the audio, video, and screen recordings are integrated and analyzed using audio/video/computer screen and text analysis software and are reviewed and triangulated [24, 44, 46]. Then, audio “think aloud” data are transcribed and uploaded to the audio/video/computer analysis software. Transcripts are annotated with video data of user actions involving interactions with the HIS and screen recording data. In clinical simulations, the data are coded to identify the occurrence of errors in the form of either “slips” (near misses) and “mistakes” (errors) [1, 31]. In the next phase of our research, we compare the results of usability inspection of the HIS using our developed heuristics to assess the predictive capability of the safety heuristics using clinical simulations. This involves summarizing the observed errors (e.g., slips and mistakes made by users interacting with HIS) during clinical simulations and comparing their nature and frequency with those predicted by the heuristics developed in Phase 1 (based on the literature and expert panel). We have employed a similar approach in the analysis of a number of HISs to assess the predictive capability of error coding schemes (based on heuristic categories) for detecting technology-induced error involving electronic patient record systems and medication administration systems [1].

## 4. Discussion and Conclusions

There are two aspects of the methodology described in this paper that make it significant. First, the paper describes a new method for developing a set of evidence-based, safety heuristics that can be used to evaluate the safety of HIS interface designs used in complex, dynamic, and uncertain work settings. Few heuristics are specifically designed around safety of HIS user interfaces. These heuristics can be used by software developers to design safe HIS interface designs for complex and dynamic work settings such as those found in healthcare. As well, these heuristics can be used by human factors experts working in the field to identify potential sources of technology-induced error in systems that are currently being used (in order to prevent any future errors that can lead to human death, disability, and injury). Second, from a methodological perspective, the paper describes the development of an approach that links several different but complementary methodologies in a novel way to develop and empirically test HIS interface design heuristics (i.e., the use of a systematic review to inform heuristic development by a panel of human factors experts and the use of inspections of several IS interface designs by human factors experts followed by clinical simulation testing to determine the ability of the heuristics to predict HIS interface design safety issues). 

The authors are currently conducting tests of heuristics on the static (e.g., fixed user interface features) and dynamic features (e.g., aspects of the user-system dialogue and interaction in carrying out real tasks) of user interface designs [23, 30]. Further research is needed to develop evidence-based (i.e., research based) heuristics and to assess the ability of such heuristics to predict those static and dynamic features of an interface design that may facilitate technology-induced errors [30]. This testing needs to be done under conditions that replicate real-world environments in response to examples of situations that users typically encounter (i.e., human-in-the-loop testing) [8]. Furthermore, there is a need to extend this work to create a set of safety heuristics and a methodology for evaluating HIS interface safety that is effective in predicting static and dynamic features of interfaces that induce technology-induced errors [8, 23, 24, 30]. Simulation testing needs to take place for this to occur, where predictions made from applying heuristics are evaluated by observing users interacting with systems in complex environments and using clinical simulations. Such testing of safety heuristics involving user interface designs from healthcare will be key to the development and validation of heuristics for this complex, dynamic environment, where there is urgency and ambiguity associated with user decision making, and where it is necessary to identify those sources of technology-induced error that may arise from human-computer interaction before widespread system implementation [1, 46]. Ideally, such use of evidence-based heuristics will be key to detecting user interface features or functions that could potentially lead to such errors prior to workers' use of HIS in the real world. This is especially necessary as errors arising from HIS use in complex environments may lead to human harm (i.e., death, disability or injury) [6, 31, 32], and the costs of addressing the unsafe software are much lower to society than the costs of treating individuals who are harmed by unsafe software [14]. 

Our work indicates that the evaluation of such heuristics requires testing under realistic conditions that can be provided using simulation methods. Such an approach can be used in the empirical validation of HIS that are currently being used. From a theoretical perspective, the extension of work done in this area of human factors contributes to the development and validation of safety heuristics using clinical simulations. In summary, in this paper we outline a new methodology for developing evidence-based safety heuristics for HIS interface design and a methodology for developing and testing such evidence-based safety heuristics using clinical simulations. The full methodology (i.e., systematic review, expert panel development, inspections, and clinical simulation as applied to validation of safety heuristics testing) can be used in improving system safety. 

## Figures and Tables

**Table 1:** Usability themes and corresponding number of heuristics developed in each theme.

Usability theme	Definition for each theme	Number of heuristics developed in each area
Workflow	Workflow issues deal with process issues arising from HIS use	10
Content	Content issues arise from poor quality information in the HIS	14
Safeguards	Safeguard issues are specific to the presence or absence of decision supports that prevent medical errors	8
Function	Functional issues deal with concerns arising from how a HIS functions	6

**Table 2:** Examples developed safety heuristics organized by usability theme.

Usability theme	Example of developed heuristic
Content	“System clearly displays the date and time the medication was updated” [23]
Workflow	“System accommodates clinician physical activities” [23]
Functional	“System allows for linkages between medication ordering, administration, and discontinuation procedures” [23]
Safeguards	“System checks for duplicated medications” [23]
